# OLDER ADULTS PURSUE ENCORE CAREERS IN RESEARCH TO PROMOTE INCLUSION OF OLDER ADULT PEERS IN CLINICAL TRIALS

**DOI:** 10.1093/geroni/igae098.0411

**Published:** 2024-12-31

**Authors:** Kathryn Nearing, Leslie Wright, Christin Williamson, Cory Sedey-Seitz

**Affiliations:** University of Colorado Anschutz Medical Campus, Denver, Colorado, United States; Kaiser Permanente Colorado, Aurora, Colorado, United States; Clinical Research Administration, Office of Regulatory Compliance, Aurora, Colorado, United States; Clinical Research Support Center, University of Colorado Anschutz Medical Campus, Aurora, Colorado, United States

## Abstract

We created an innovative pathway to encore careers in research for older adults at the University of Colorado Anschutz Medical Campus. Our program trains older adults from the community (average age 68; range: 56-82) for specialized community-facing and participant-centered roles on clinical trial teams to increase the recruitment and retention of older adult peers, as well as other underrepresented populations, in clinical trials. Older Adult Research Specialists’ roles include: 1) developing effective communication and outreach strategies to engage older adults from diverse backgrounds in research; 2) recruiting and consenting participants for trials; 3) supporting retention by identifying barriers to study participation and connecting study participants with needed resources to address social needs; 4) educating research teams regarding key considerations for including older adults in research, anchored by the Geriatric 5Ms, to inform trial design and implementation; and, 5) catalyzing research innovations to increase representation in, and the relevance and translational potential of, research. OARS prepare for their essential roles on clinical trial teams by completing a 14-week, 140-hour, virtual, competency-based training program. Health Navigation training is the foundation. To date, we have trained four cohorts (43 graduates). OARS are making the research workforce and associated teams more age-inclusive in the Division of Geriatric Medicine, Department of Neurology, Department of Physical Medicine and Rehabilitation Sciences, Center for Inclusive Design and Engineering, and at our healthcare affiliate, the Rocky Mountain Regional VA Medical Center. By promoting intergenerational learning, encore careers and research innovation, OARS are advancing our priorities as an Age-Friendly University.

